# Glioma immunotherapy enhancement and CD8-specific sialic acid cleavage by isocitrate dehydrogenase (IDH)-1

**DOI:** 10.1038/s41388-023-02713-7

**Published:** 2023-05-09

**Authors:** Ryan Cordner, Michelle Jhun, Akanksha Panwar, HongQiang Wang, Nicole Gull, Ramachandran Murali, Joseph H. McAbee, Armen Mardiros, Akane Sanchez-Takei, Mia W. Mazer, Xuemo Fan, Emmanuel Jouanneau, John S. Yu, Keith L. Black, Christopher J. Wheeler

**Affiliations:** 1 Department of Neurosurgery, Maxine Dunitz Neurosurgical Institute, Cedars-Sinai Medical Center, Los Angeles, CA USA; 2 Department of Biomedical Sciences, Center for Bioinformatics and Functional Genomics, Cedars-Sinai Medical Center, Los Angeles, CA USA; 3 Department of Biomedical Sciences, Research Division of Immunology, Cedars-Sinai Medical Center, Los Angeles, CA USA; 4 Department of Pathology and Laboratory Medicine, Cedars-Sinai Medical Center, 8700 Beverly Blvd., Los Angeles, CA USA; 5 Present Address: Department of Microbiology and Molecular Biology, Brigham Young University, UT Provo, USA; 6 Present Address: Surgical Neurology Branch, National Institute of Neurological Disorders and Stroke, NIH, Bethesda, MD USA; 7 Present Address: A2 Biotherapeutics, Agoura Hills, CA USA; 8 Present Address: Department of Neurosurgery, Neurological Hospital and INSERM 842 Research Unit, Claude Bernard University, Lyon, France; 9 Present Address: International Brain Mapping Foundation, Society for Brain Mapping & Therapeutics, 860 Via De La Paz, Suite E-1, Pacific Palisades, CA USA; 10 Present Address: StemVax Therapeutics (subsidiary of NovAccess Global), 8584 E. Washington St. #127, Chagrin Falls, OH USA; 11 Present Address: T-Neuro Pharma, PO Box 781, Aptos, CA USA

**Keywords:** Tumour immunology, Prognostic markers

## Abstract

The promise of adaptive cancer immunotherapy in treating highly malignant tumors such as glioblastoma multiforme (GBM) can only be realized through expanding its benefits to more patients. Alleviating various modes of immune suppression has so far failed to achieve such expansion, but exploiting endogenous immune enhancers among mutated cancer genes could represent a more direct approach to immunotherapy improvement. We found that Isocitrate Dehydrogenase-1 (IDH1), which is commonly mutated in gliomas, enhances glioma vaccine efficacy in mice and discerns long from short survivors after vaccine therapy in GBM patients. Extracellular IDH1 directly enhanced T cell responses to multiple tumor antigens, and prolonged experimental glioma cell lysis. Moreover, IDH1 specifically bound to and exhibited sialidase activity against CD8. By contrast, mutant IDH1R132H lacked sialidase activity, delayed killing in glioma cells, and decreased host survival after immunotherapy. Overall, our findings identify IDH1 as an immunotherapeutic enhancer that mediates the known T cell-enhancing reaction of CD8 desialylation. This uncovers a new axis for immunotherapeutic improvement in GBM and other cancers, reveals novel physiological and molecular functions of IDH1, and hints at an unexpectedly direct link between lytic T cell function and metabolic activity in target cells.

## Introduction

Adaptive immunotherapy is emerging as a promising treatment for a variety of cancers, including highly malignant tumors such as Glioblastoma multiforme (GBM). Most adaptive immunotherapies reliably activate T cells that can destroy tumors, and have yielded impressive clinical benefits in a variety of malignancies. For example, unusual 3- to 5-year survivors have been reported in up to 40% of GBM patients enrolled in dendritic cell (DC) vaccine trials conducted by us and others [1, 2]. Nevertheless, immunotherapy typically fails to benefit most patients with any type of tumor. Various immune suppressive factors have been implicated in clinical failure of cancer immunotherapy. These include immunosuppressive cytokine production [3], T cell death or regulatory receptor ligation [4], and immune checkpoint induction [5]. Indeed, overcoming immune suppression is the basis for potent immunotherapies such as immune checkpoint inhibition, that nevertheless fail to benefit most patients due to high tumor burden and other factors [6, 7]. It is thus critical to determine alternate factors mediating the clinical success of adaptive immunotherapies.

Endogenous immune enhancing factors could supplement immunosuppressive inhibition to further improve immunotherapy. Direct evidence for such factors comes from studies of p53, whose activation in tumors counteracts immunosuppression and bolsters anti-tumor immunity [8, 9]. Several other tumor suppressor genes have been shown to modulate T cell activity as well [9]. Immune enhancer candidates may thus be found among classical tumor suppressors, or similar genes that are frequently inactivated in malignancies.

Prior studies indicate that local desialylation enhances beneficial anti-tumor T cell activity [10]. This can in theory occur via immunogenic modification of tumor peptide antigens, or by removal of sialic acid from the stalk region of the T cell coreceptor, CD8 [11–13]. In practice, the latter appears to more prominently enhance anti-tumor T cell activity by increasing avidity of CD8 for peptide-MHC I on target cells [12, 13]. Such enhancement has only been demonstrated through treatment of T cells with exogenous sialidases such as *Vibrio cholerae* neuraminidase (VCN), however, which ultimately promotes T cell death via nonspecific desialylation. Endogenous factors that physiologically enhance anti-tumor T cell activity through CD8 desialylation are not known, in part because defined sialidases are neither highly expressed, nor found frequently mutated, in tumors including GBM [14]. We therefore examined whether other genes mutated in gliomas had immune enhancing and/or desialylation potential.

Isocitrate dehydrogenase (IDH)-1 is frequently mutated in low-grade gliomas and secondary GBM, as well as in blood (AML) and liver tumors [15, 16]. Tumor-associated IDH1 mutations confer the novel ability to produce the oncometabolite, 2-hydroxyglutarate (2HG), but also impair anti-tumor immunity in gliomas [17]. While 2HG production contributed to such impairment in an experimental glioma, this raised the unexplored possibility that wild-type IDH1 is a tumor-derived immune enhancing factor. A report that NADP^+^-dependent IDH shares active site structural similarities with microbial neuraminidase linked this possibility to desialyation [18], and prompted us to examine IDH1’s influence on T cell activity.

We examined the impact of mutated and wild-type IDH1 on host survival after vaccine immunotherapy, in both glioma-bearing mice and GBM patients. Our results suggested that IDH1 possesses immune enhancing activity separate from its metabolic function. Indeed, IDH1 over-expression in glioma cells prolonged killing by cytolytic T cells (CTL), and extracellular IDH1 enhanced T cell responsiveness to tumor peptide-MHC. IDH1 but not IDH1R132H mediated novel sialic acid-cleaving activity against glycoproteins, with preference for the CD8 co-receptor at limiting concentration. Taken together, our findings demonstrate surprising immune potentiating activity of IDH1. These findings have significant implications for the function of IDH1 and its mutants in cancer and possibly other diseases, their use as immunotherapeutic biomarkers and modulators, and the linkage between target cell metabolism and CTL lysis.

## Results

### IDH1R132H confers age-dependent survival to glioma hosts

**Fig. 1:**
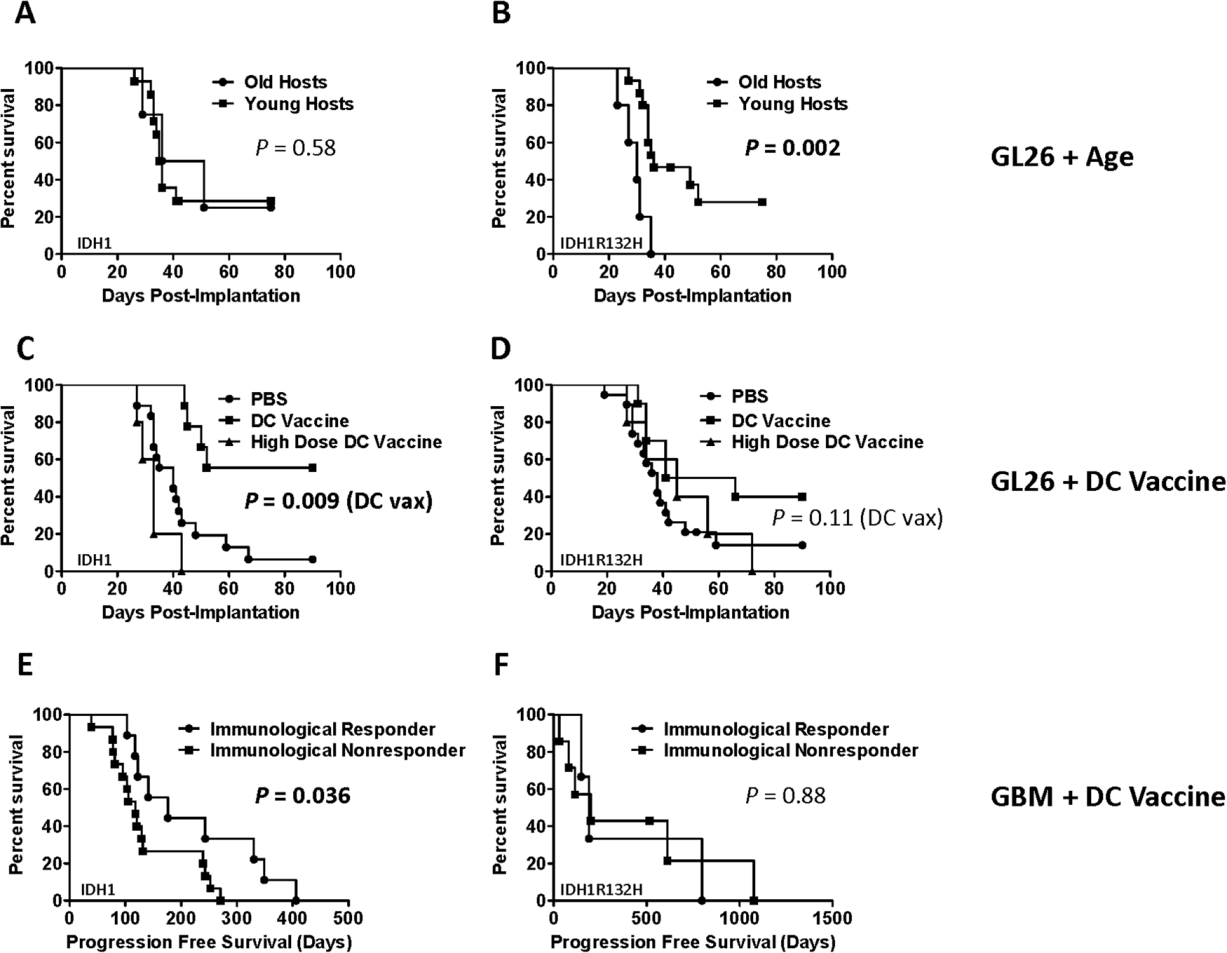
IDH1R132H tumors exhibit vaccine resistance in humans and mice. **A** In C57BL/6 mice implanted with GL26-IDH1 (*n* = 14 young, *n* = 4 old), differences in survival did not reach significance (*p* = 0.5757, Log-rank). **B** In C57BL/6J mice implanted with GL26-IDH1R132H (*n* = 15 young, *n* = 5 old), survival of old vs. young was significantly different (*p* = 0.002, Log-rank test). **C**, **D** C57BL/6 mice were implanted with GL26-IDH1, or with GL26-IDH1R132H, and treated with either PBS (*n* = 14-15), low dose (therapeutic) DC vaccine (*n* = 9-10), or high dose (detrimental) DC vaccine (*n* = 5). The survival benefit associated with vaccination (*p* < 0.01 in GL26-IDH1 by log-rank) is abrogated by the R132H mutation (*p* > 0.09 in GL26-IDH1R132H by log-rank). **E**, **F** Comparison of progression-free survival between IDH1 mutation-negative, or IDH1R132H GBM patients that were either immunological responders (*n* = 9 for IDH1; *n* = 3 for IDH1R132H) or immunological non-responders (*n* = 15 for IDH1; *n* = 7 for IDH1R132H), after administration of autologous tumor lysate-pulsed DC vaccine. For IDH1 patients, *p* = 0.036; *p* = 0.88 for IDH1R132H patients, by Log-Rank.

**Fig. 2:**
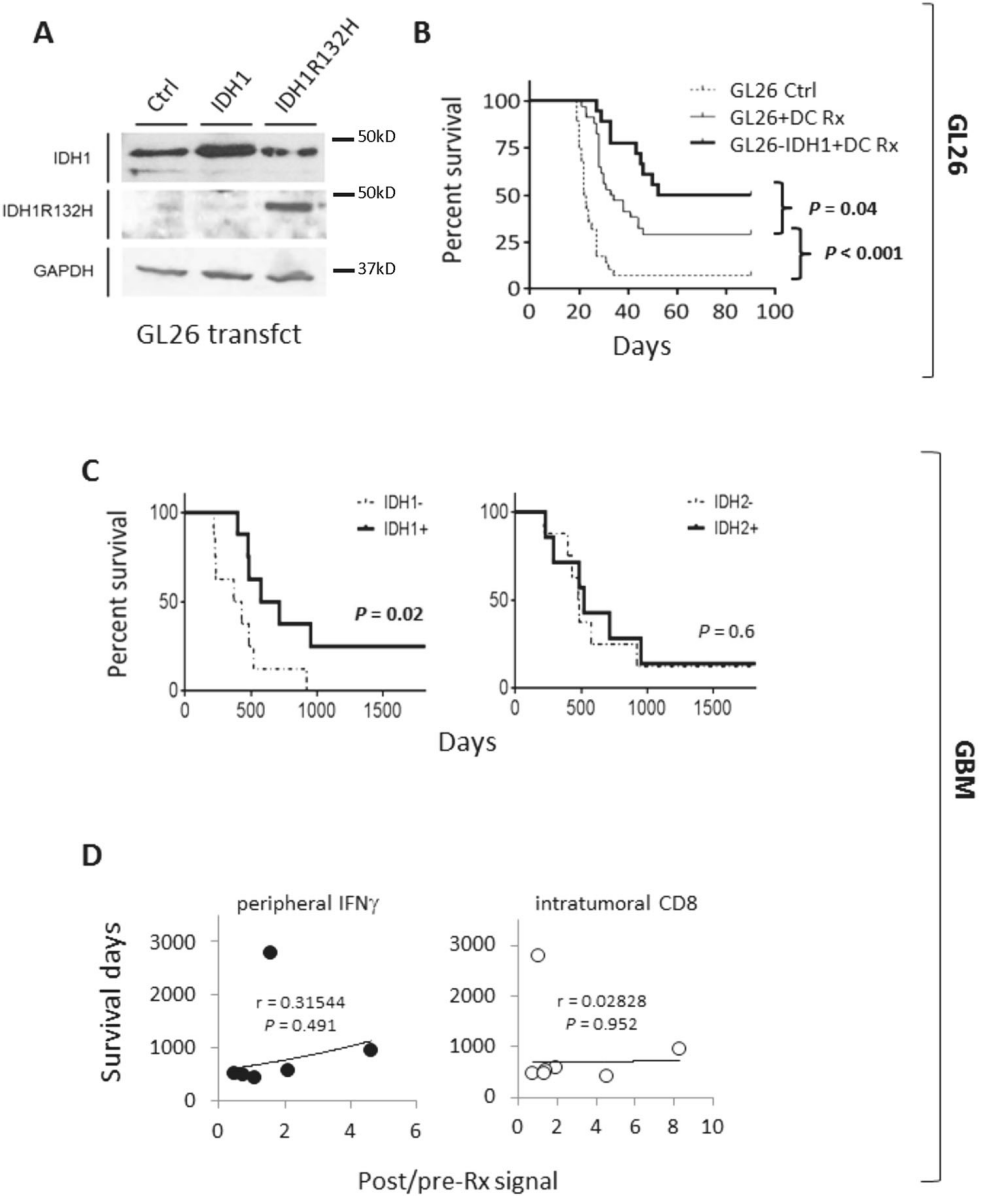
Wild-type IDH1 expression and clinical immunotherapy metrics in GBM patients. **A** IDH1, IDH1R132H, and control (GAPDH) protein expression in GL26 transfectants prior to implantation. **B** IDH1 transfection into GL26 prolongs C57BL/6 (B6) host survival after DC vaccine therapy. **C** Newly diagnosed GBM from 16 DC vaccine therapy patients were subjected to microarray expression analysis before and after treatment (*n* = 10), or genomic sequencing (*n* = 6), and stratified by retention and loss of IDH1/IDH2 expression or gene copies (copy number loss = CNL), respectively. IDH1 CNL tumors (*n* = 3) were excluded from IDH2 analysis, and IDH2 CNL tumors (*n* = 1) were excluded from IDH1 analyses. Patients retaining IDH1 but not IDH2 exhibited significantly longer overall survival. Survival differences between IDH1-loss and -retained groups was increased when GBM without post-treatment CD8 infiltration were excluded from analysis (*n* = 4 & 8, respectively; 372 vs. 716 days median; *P* = 0.006 by Log-Rank). **D** Post-vaccine IFNγ production by CD8 T cells, and CD8 signal within tumor tissue (Affymetrix HG-U133+2 probeset 205758_at), both failed to significantly correlate with patient survival.

To examine the impact of IDH1 mutation and activity on age-dependent glioma outcomes, we implanted GL26 transfected with either wild-type IDH1 or IDH1R132H into young and old syngeneic C57BL/6 (B6) mice. Wild-type IDH1 transfectants (GL26-IDH1) exhibited no significant survival difference in young and old hosts, as we similarly reported for parental GL26 (Fig. 1A) [19]. IDH1R132H transfectants (GL26-IDH1R132H), however, exhibited significantly decreased survival in aged relative to young hosts (18–25 months vs. 6–8 weeks, respectively; Fig. 1B). GL26-IDH1R132H and GL26-IDH1 expressed IDH1R132H and over-expressed wild-type IDH1 protein, respectively (Fig. 2A). The dependence of age-dependent survival on CD8 T cell activity in the GL26 model [10, 20] thus suggested a specific impact of IDH1R132H protein on anti-tumor T cells. Since IDH1R132H is expressed only in tumors, this could further exacerbate intrinsic deficits in T cell antigen responses with aging. Such deficits were readily detectable against a number of tumor-associated epitopes (Supplementary Fig. S1A).

### IDH1R132H diminishes vaccine survival benefits in mouse and human gliomas

To address the impact of IDH1 mutation on anti-tumor T cell activity, we examined how IDH1 and IDH1R132H transfection impacted GL26 progression in DC vaccine-treated syngeneic (C57BL/6; B6) hosts. GL26-IDH1R132H and GL26-IDH1 exhibited identical survival in T cell-deficient B6.Foxn1 nude mice (*P* = 0.95; data not shown), and in untreated young B6 mice (Fig. 1A, B). Treatment with 50,000, GL26 tumor lysate-pulsed DC 2.4 cells in young B6 hosts (low-dose therapeutic DC vaccine) resulted in increased peripheral CD8 T cells reactive to Trp-2, the dominant CD8 T cell epitope in GL26 [10], in both GL26-IDH1 and GL26-IDH1R132H hosts (Supplementary Fig. S1B). Nevertheless, DC vaccination significantly prolonged survival in GL26-IDH1, but failed to do so in GL26-IDH1R132H hosts (Fig. 1C, D). Thus, IDH1R132H in gliomas impaired immunotherapy success without increasing anti-tumor CD8 T cells, as has been shown in separate glioma models [17].

To determine if the impact of IDH1R132H on glioma immunotherapy was uniformly pro-oncogenic, and thus related to its oncometabolite (i.e., 2HG) production, we examined whether it differentially affected host survival upon exposure to detrimental T cell activity. GL26-IDH1 host survival was decreased by administration of a very large vaccine dose (2 × 10^7^ GL26 tumor lysate-pulsed DC 2.4 cells), reminiscent of other models of immunotherapy-induced immunosuppression [21, 22] (Fig. 1C). GL26-IDH1R132H nullified this effect, restoring survival to that seen in untreated hosts (Fig. 1D). Thus, IDH1R132H-mediated modulation of GL26 host survival depended on the specific impact of immune intervention, decreasing survival with beneficial immunotherapy and increasing it with detrimental vaccination. These findings are inconsistent with a dominant influence of IDH1R132H oncometabolite production in this model, and instead suggest the mutation may inactivate an immune-potentiating function of wild-type IDH1.

To further explore this, we examined immune response metrics in human GBM, focusing first on our own immunotherapy patients. As in previous trials, immune responding patients with wild-type IDH1 (those exhibiting ≥50% increased IFNγ tumor antigen response post-vaccine without IDH1R132H) showed significantly increased progression-free survival (PFS) after DC vaccination (Fig. 1E). In contrast, GBM patients with IDH1R132H exhibited no increase in PFS after DC vaccination (Fig. 1F), despite unaltered immune response magnitudes (Supplementary Fig. S2A). Moreover, all patients with IDH1R132H exhibited either endogenous or vaccine-induced immune responses, whereas approximately a third of those lacking the mutation did not (Supplementary Fig. S2B). Thus, IDH1R132H is associated with impaired vaccine clinical efficacy in mice and patients, yet is consistently associated with anti-tumor T cell responses.

### Low grade gliomas and GBM with IDH1 mutation have diminished CD8 T cell activity

Reduction of DC vaccine benefits by IDH1R132H despite systemic T cell responsiveness suggests local impairment of anti-tumor T cells. We thus examined the expression of T cell and other genes by IDH1-mutated and IDH1-wild-type gliomas in The Cancer Genome Atlas (TCGA). Low-grade gliomas with IDH1R132H exhibited significantly lower T cell effector gene expression than those without the mutation (Supplementary Fig. S3A–C). Expression of innate and other adaptive immune genes, including GFAP for activated astrocytes and IBA-1 for microgliosis, were comparable between IDH1R132H and IDH1-wild-type gliomas (Supplementary Fig. S4A–E). Similar trends were observed in GBM, but statistical significance was not reached with the smaller numbers of IDH1R132H tumors in that cohort (Supplementary Figs. S3D–F, S4F–J). Expression of most CD4 T subset-associated genes [23] was also not significantly impacted by IDH1 mutation in low grade gliomas (Supplementary Fig. S4K). These data expand on recent reports associating IDH1R132H with reduced CD8 T cell gene expression in TCGA gliomas [17], which implicated 2HG-mediated inhibition of CXCL10 production in lower T cell recruitment and metabolic reprogramming in CD8 T cells leading to anti-glioma immunosuppression [24, 25]. We did not observe reduced GFAP and IBA-1 expression, however, which should also be impacted by CXCL10 inhibition [26]. Thus, data from human GBM corroborate that IDH1R132H may impair anti-tumor T cells independent of 2HG-mediated effects.

### IDH1 over-expression enhances DC vaccine in GL26, and its retention predicts GBM vaccine success

To directly test whether wild-type IDH1 possesses an immune enhancing function eliminated by IDH1R132H, we examined post-vaccine host survival in IDH1-over-expressing GL26-IDH1 (Fig. 2B) in greater detail. Host survival more than doubled after DC vaccination in GL26-IDH1 compared to untransfected and control-transfected GL26 (median 52 days; Fig. 2B and Supplementary Fig. S5, respectively), rendering a majority of hosts long-term survivors. Unmodified GL26 tumors that grew intracranially despite therapeutic vaccination also exhibited decreased expression of IDH1 RNA on microarray analysis, relative to GL26 growing in brains of either unvaccinated or T cell-deficient mice (Supplementary Fig. S6A). Thus, IDH1 over-expression increased GL26 vaccine success, while vaccine failure was accompanied by enrichment of IDH1-lo GL26 cells, possibly due to their less efficient destruction by T cells.

In vaccinated GBM with increased CD8 tumor signal post-treatment (i.e., local treatment response), IDH1 DNA or RNA loss predicted significantly shorter patient survival (Fig. 2C). GBM with low IDH1 expression before DC vaccination also failed to increase CD8 signal after vaccine therapy (Supplementary Fig. S6B). By contrast, IDH2 retention was not associated with longer patient survival after vaccination (Fig. 2D), nor was IFNγ production by peripheral T cells, or increased CD8 in tumors after treatment (Fig. 2D). Together, these findings suggest that retention of wild-type IDH1 in gliomas enhances clinical success of T cell-activating immunotherapy, whereas IDH1 loss impairs local anti-tumor T cell responses and leads to immunotherapy failure. The contrasting finding with IDH2, which catalyzes the identical metabolic reaction as IDH1 in cells, further emphasizes that IDH1 may enhance T cell activity independent of its known enzymatic function.

### IDH1R132H diminishes and IDH1 enhances glioma lysis by CD8^+^ cells

**Fig. 3:**
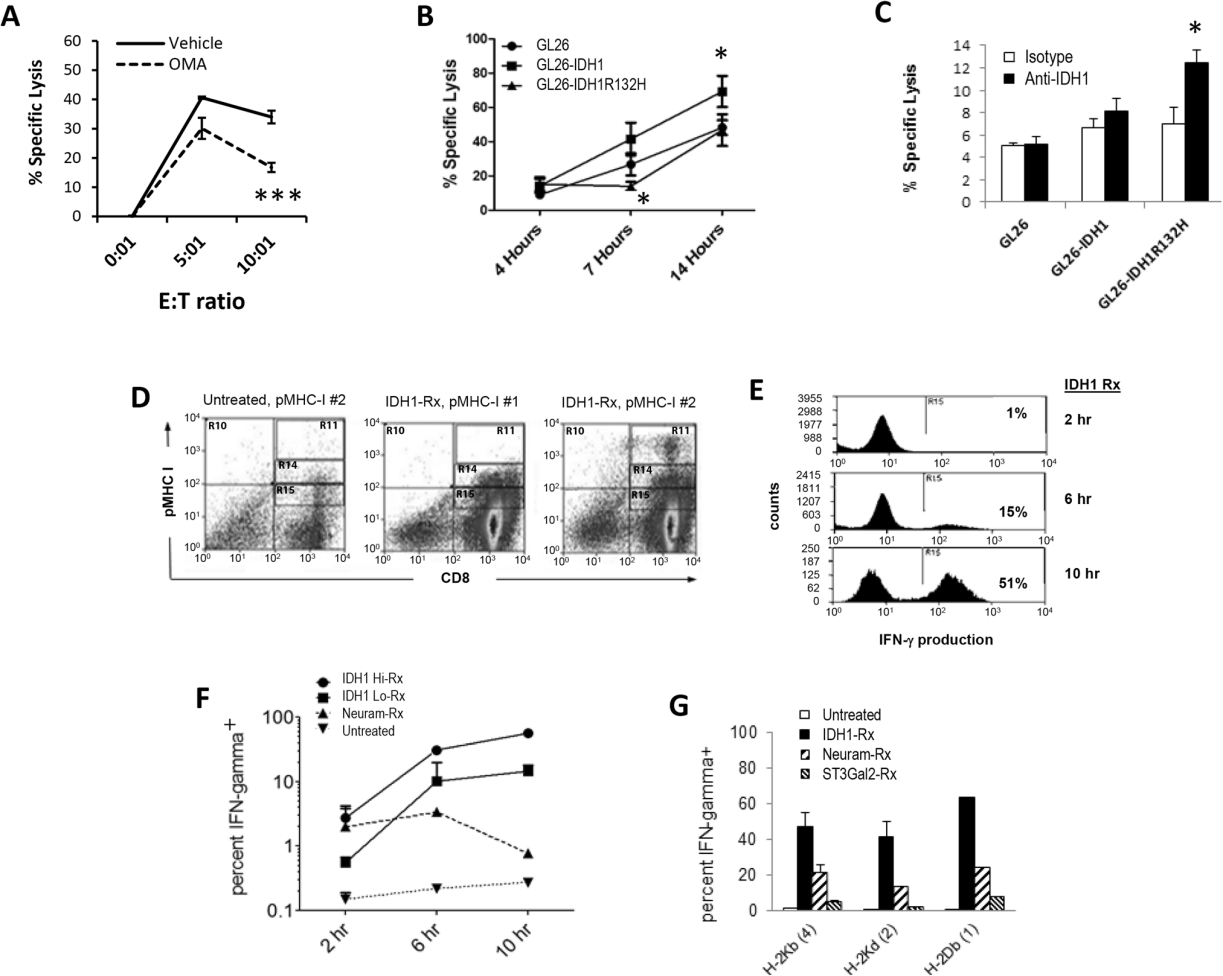
IDH1 treatment increases multimer binding and cytokine production in CD8 T cells. **A** GL26 target lysis by an H-2K^b^ reactive T cell hybridoma effectors after 7 h, +/−5 mM oxalomalic acid (OMA); *p < 0.05, ***p < 0.005. **B** Parental GL26, IDH1 and IDH1R132H transfectant lysis over time by H-2K^b^ reactive hybridoma effectors (E:T ratio 10:1); *p < 0.05. **C** Anti-IDH1 antibody increased early lysis of GL26-IDH1R132H targets by CTL from GL26-vaccinated mice. Anti-IDH1 had no impact on CTL lysis of 7 and 16 h targets (4 h with E:T = 10:1 E:T is shown; *p < 0.05). **D** Enhanced binding of IDH1-treated CD8 T cells to pMHC I dextramers. **E** IDH1-treated C57BL/6 (B6) mouse CD8 T cells producing IFNγ expand following stimulation with an H-2K^b^/TVSEFLKL Survivin dextramer + anti-CD28 antibody. **F** B6 CD8 T cell IFNγ production over time stimulated by H-2K^b^/SVYDFFVWL Trp-2 dextramer + anti-CD28, after treatment with IDH1, neuraminidase, or control (Untreated or No Rx = [dextramer+anti-CD28], no IDH1-treatment). **G** 10-h compilation of distinct pMHC I dextramer + anti-CD28 stimulation.

To further examine requirements for IDH1-mediated effects on T cell activity, we examined T-mediated killing of GL26 with and without oxalomalic acid (OMA), a competitive inhibitor of IDH1 substrate binding and catabolic activity [27–29]. OMA significantly impaired GL26 killing by a CD8^+^ T cell hybridoma reactive to H-2K^b^ (HTB-156.7) at 10:1 effector:target cells (E:T ratio; Fig. 3A). This suggests that IDH1 substrate binding and/or catabolic activity is necessary for optimal T cell lysis, but whether IDH1 was tumor-derived was unclear. We thus compared HTB-156.7 killing of GL26 to that of GL26-IDH1 and GL26-IDH1R132H. In addition to expressing transfected genes appropriately (Fig. 2A), GL26-IDH1 and GL26-IDH1R132H exhibited unique alterations in lipids under glucose-starvation and recovery conditions, respectively, confirming distinct metabolic alterations (Supplementary Fig. S7) [30–32]. H-2K^b^ antigen expression was also unaltered by the transfections (Supplementary Fig S8A), and their sensitivity to cell death induced by the caspase 8 agonist, imidazole, was comparable (Supplementary Fig. S8B), consistent with prior studies [33, 34]. Exogenous 2-hydroxyglutarate (2HG) did not significantly alter overall effector function (IFNγ production; Supplementary Fig. S9A) or proliferation (Supplementary Fig. S9B), also consistent with prior studies [35]. Despite this, lysis of GL26-IDH1R132H targets was delayed after 4 h compared to untransfected GL26, whereas GL26-IDH1 lysis was enhanced at later time points (Fig. 3B). This suggests that intracellular IDH1 and IDH1R132H in tumors enhance and inhibit T cell activity, respectively. Because IDH1 is not normally secreted, this may occur through the release of active cytoplasmic IDH1 or IDH1R132H from CTL-lysed or otherwise ruptured tumor cells. Indeed, ample catalytic activity of IDH1/NADP^+^ was present in supernatants of lysed tumor cells (Supplementary Fig. S8C). More directly, extracellular anti-IDH1 antibody significantly increased GL26-IDH1R132H killing by CTL from tumor-vaccinated mice after 4 h (Fig. 3C). Together, these findings broadly support the notion that glioma killing by CTL is modulated by cytoplasmic IDH1 proteins released from lysed tumor cells.

### Extracellular IDH1 enhances CD8 T cell reactivity

IDH1 could conceivably enhance CTL activity by altering a distinct immunoactive substrate. Sialic acid may represent such a substrate, given the reported structural similarity between NADP^+^-dependent IDH and a microbial sialic acid-cleaving enzyme (neuraminidase/sialidase) [18]. Indeed, removal of sialic acid from o-linked glycans on the CD8β stalk by *Vibrio cholerae* neuraminidase (VCN) enhances coreceptor binding to peptide-MHC I (pMHC-I), and increases T cell responsiveness to pMHC-I by mouse and human T cells [10–13]. We thus examined whether IDH1 outside the cell similarly affects CD8 T cell responsiveness to pMHC-I multimer stimulation. Treatment of mouse CD8 T cells with IDH1 and NADP^+^ enhanced binding to tumor pMHC-I (Fig. 3D). More importantly, IDH1 treatment of CD8^+^ HTB-156.7 hybridoma cells led to its increased IFNγ production in response to pMHC-I stimulation (Fig. 3E), which was sustained over time as VCN-treated cells died, and increased with higher IDH1 (Fig. 3F). This was observed with several pMHC-I multimers (Fig. 3G). These results suggest that, like VCN, extracellular IDH1 increases antigen activation of mouse T cells. Moreover, IDH1-mediated response enhancement was independent of T cell epitope specificity, consistent with an impact on CD8 [10, 13].

Treatment of human CD8 T cells treated with exogenous IDH1/NADP^+^ led to a marked increase in the proportion of cells binding MAGE-1/HLA multimers (Supplementary Fig. S10A). This multimer-bound population also exhibited increased binding to peanut agglutinin (PNA), a lectin that binds quantitatively to desialylated galactosyl (β-1,3) N-acetylgalactosamine glycans on cell surfaces (Supplementary Fig. S10B). The PNA binding increase was prevented by excess CMP-sialic acid, consistent with competitive inhibition of desialylation. Moreover, pHLA binding was prevented by addition of anti-CD3 antibody prior to flow cytometry (Supplementary Fig. S10A), suggesting involvement of both TCR and CD8. Similar augmentation of pMHC-I and PNA binding was seen in IDH1-treated mouse cells responding to a distinct tumor-associated Survivin epitope (Supplementary Fig. S10C, D). Together, these findings indicate that extracellular IDH1 directly enhances T cell activity, and that this enhancement involves T cell desialylation.

### IDH1 but not IDH1R132H removes sialic acid from glycoproteins

**Fig. 4:**
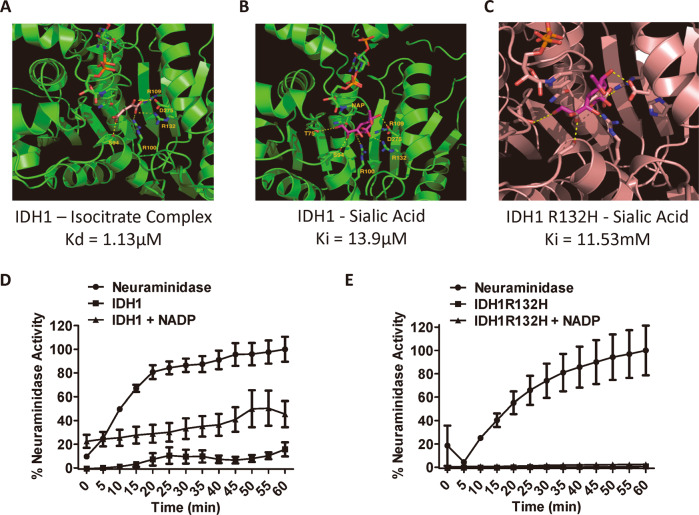
IDH1 demonstrates sialidase activity. **A** Crystal structure of isocitrate and NADP^+^ bound in the active site of IDH1. Dashed yellow lines show hydrogen bonds. **B** Predicted model of sialic acid in the active site of IDH1. **C** Predicted model of sialic acid in the active site of IDH1R132H. **D** Sialic acid release assay of IDH1 relative to *V. cholera* neuraminidase (VCN), demonstrates in vitro IDH1-mediated sialidase activity against purified CD8β monomer that is largely dependent on NADP^+^. **E** IDH1 sialidase activity against CD8β is abrogated by the IDH1R132H mutation.

The possibility that IDH1 can cleave sialic acid from glycans was first examined through computational modeling, which indicated that sialic acid can in theory bind to the catalytic site of IDH1 with a Ki of 13.9 μm. In this analysis, sialic acid binding was dependent on many of the same catalytic site residues that bind isocitrate, and was similarly stabilized by NADP^+^ (Fig. 4A, B). By contrast, IDH1R132H modeling predicted much weaker sialic acid binding, with or without NADP^+^ (Fig. 4C).

Further consistent with sialic acid-binding, IDH1-mediated production of NADPH from isocitrate + NADP^+^ was inhibited and by 6’-sialyllactose, a sialidase substrate, as well as by its known catalytic inhibitor, α-ketoglutarate (aKG; Supplementary Fig. S11A). This provided empirical evidence that IDH1 residues involved in isocitrate catalysis competitively interact with sialylated glycans. We next measured release of sialic acid from fetuin and CD8 glycoproteins by IDH1 and IDH1R132H with and without NADP^+^. Relative to VCN, IDH1 alone displayed minimal activity, but induced significant sialic acid release from CD8β with NADP^+^ (Fig. 4D). The same concentration of IDH1R132H failed to exhibit detectable sialidase activity against CD8β with or without NADP^+^ (Fig. 4E). Antibody absorption of IDH1 also decreased sialidase activity against fetuin from GL26-IDH1 lysate, whereas sialidase activity in GL26-IDH1R132H lysate was significantly lower, and slightly increased by IDH1 absorption (Supplementary Fig. S11B). These findings are consistent with substantial reduction of IDH1-mediated sialidase activity released from dead, IDH1R132H-expressing tumor cells.

**Fig. 5:**
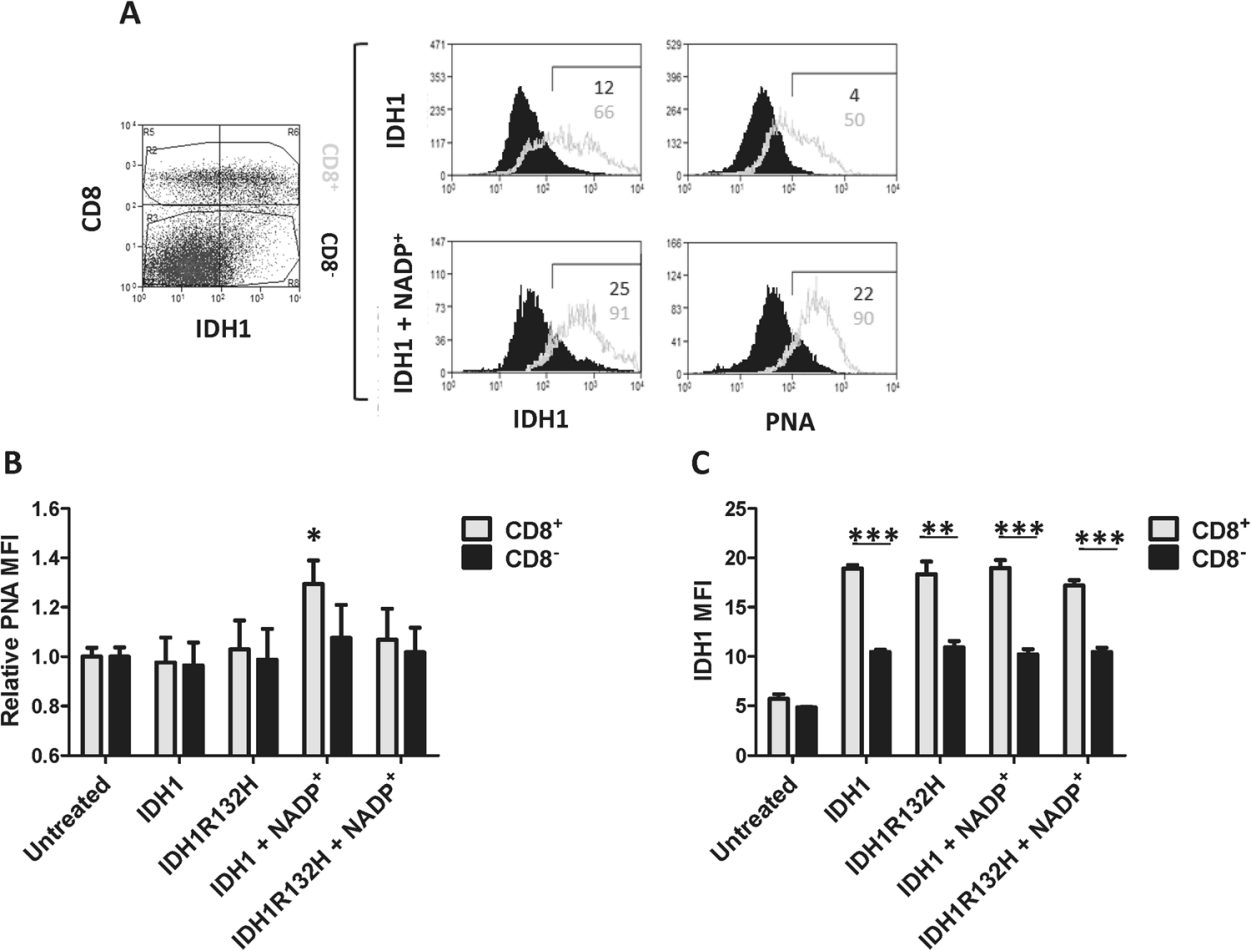
IDH1 binds to and desialylates CD8+ cells. **A** Flow cytometric analysis of splenocytes exogenously treated with FITC-labeled IDH1 demonstrates higher binding of IDH1 to CD8^+^ cells and selective desialylation of those cells. **B** Relative PNA fluorescence intensity (MFI) is increased by IDH1 only on CD8^+^ cells, and is dependent on both NADP^+^, and a wild-type R132 residue. **C** Quantification of FITC-labeled IDH1 and IDH1R132H MFI demonstrates preferential binding to CD8^+^ cells, irrespective of R132 mutation. *p < 0.05; **p < 0.01; ***p < 0.001.

To determine if IDH1 desialylates surface molecules on cells, we incubated splenocytes with exogenous IDH1, which revealed significantly more PNA binding by flow cytometry to CD8^+^ compared to CD8-negative cells in the presence of NADP^+^, whereas no such increase was observed in the absence of NADP^+^ (Fig. 5A, B). IDH1R132H did not significantly increase PNA binding to either CD8^+^ or CD8-negative cells. This suggests that NADP^+^ and IDH1, but not IDH1R132H, preferentially desialylates CD8^+^ cell surface molecules.

### IDH1 specifically binds and desialylates CD8 dimers

**Fig. 6:**
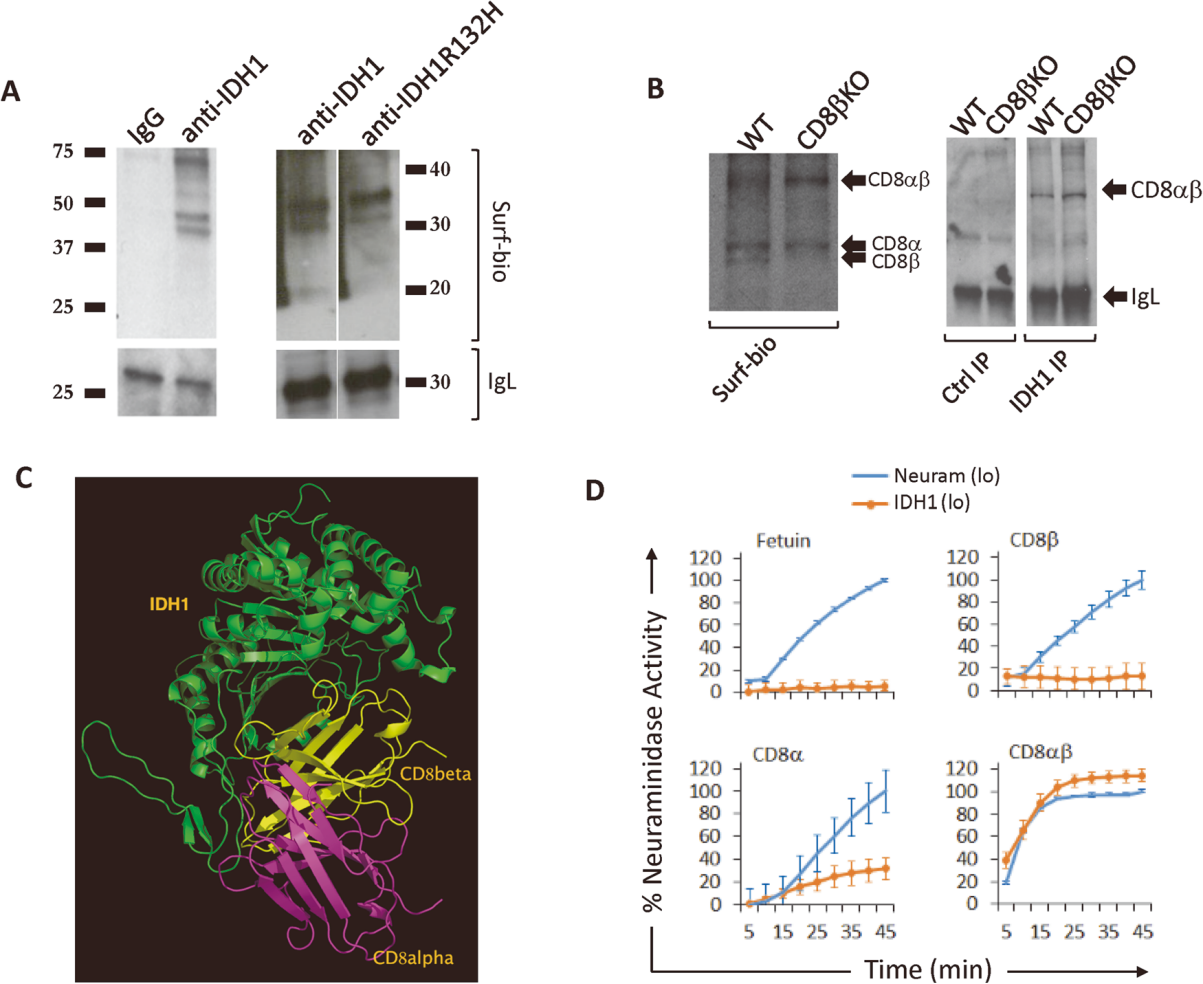
IDH1 binds to CD8. **A** Surface-biotinylated proteins coimmunoprecipitated with human and mouse IDH1, and human IDH1R132H on mouse CD8 T cells, resemble the CD8αβ heterodimer. **B** Surface-biotinylated species (left panel) and anti-CD8α Westerns (right panel), of IDH1-immunoprecipitates from wild-type and CD8β-knockout (KO) splenocyte lysates. **C** Molecular modeling predicts stable binding of IDH1 monomers to CD8αβ heterodimers. **D** Low (1 µg) concentration neuraminidase and IDH1 sialidase activity against fetuin and CD8 substrates.

We used fluorescein-labeled IDH1 and IDH1R132H in flow cytometric analyses to examine its interaction with discrete surface ligands as a potential mechanism for its preferential desialylation of CD8^+^ cells. CD8^+^ cells indeed exhibited elevated binding to fluorescein-IDH1 and fluorescein-IDH1R132H (Fig. 5A, C). We then immunoprecipitated native IDH1 from surface-biotinylated splenocyte lysates, and detected accompanying labeled proteins on blots with streptavidin. IDH1R132H protein was added to some lysates after surface labeling as well. Immunoprecipitation with either anti-IDH1 in native lysates, or anti-IDH1R132H antibody in IDH1R132H-supplemented lysates, pulled down a doublet of 38–45 kDa under reducing conditions, frequently accompanied by a larger 70 kDa species (Fig. 6A). Since this mobility was consistent with the disulfide-linked CD8αβ heterodimer, we repeated immunoprecipitations on surface-biotinylated splenocytes from CD8β-deficient mice, which revealed loss of the fastest migrating species as expected of CD8β (Fig. 6B, left panel). We then blotted and probed IDH1 immunoprecipitates of wild-type and CD8β-deficient lysates with anti-CD8α antibody, which identified a band at 70 kDa not present in control lysates (Fig. 6B, right panel; note: lower bands obscured by immunoglobulin signal). This confirmed that IDH1 specifically binds to both CD8αα and CD8αβ dimers. Indeed, molecular modeling predicted binding of monomeric IDH1 to CD8αβ dimers based on topological complementarity (Fig. 6C).

Specific binding to CD8 dimers could allow IDH1 to specifically desialylate CD8 under physiological conditions. Accordingly, low concentration IDH1/NADP^+^ desialylated CD8α as well as CD8αβ mixtures, which both form dimers. Indeed, CD8αβ desialylation by IDH1/NADP^+^ was even more efficient than by VCN. By contrast, similar concentrations of other substrates, including fetuin and isolated CD8β monomers, were not efficiently desialylated under such conditions (Fig. 6D). Thus, binding and desialylation by limiting amounts of IDH1/NADP^+^ was selective for CD8 dimers, and CD8αβ heterodimers particularly.

## Discussion

In this study, we demonstrate that IDH1 over-expression increases survival time and survivor numbers following vaccine immunotherapy in a glioma model, GL26. This model is similar but completely distinct from the better studied GL261 [36–39]. In this context, GL26 is susceptible to immunotherapy [10, 40, 41], exhibits similar genetic alterations as GBM after vaccine therapy [19], and recapitulates GBM-like T cell-mediated synergy with chemotherapy [19, 42], making it a uniquely relevant model of glioma immunotherapy. Moreover, IDH1 retention distinguished long from short survivors among immune-responsive GBM patients after therapeutic vaccination, and low IDH1 expression and IDH1 mutation were associated with immunological and clinical failure of this treatment. Recent reports show that inhibition of 2-hydroxyglutarate (2HG) production also potentiates glioma immunotherapy [17, 24, 25]. Such inhibition is relevant to IDH1-mutated gliomas, which are typically low-grade tumors and rarely seen among GBM. By contrast, wild-type IDH1 is ubiquitously expressed within cells, and exhibited T cell-modulating activity as an extracellular protein. Thus, extracellular IDH1 within tumors has broad potential to guide patient selection, predict success, and perhaps enhance clinical efficacy of immunotherapy for gliomas. Moreover, IDH1 treatment enhanced CD8 T cell responses to several antigens shared by a variety of tumor types. Thus, we believe IDH1 may similarly modulate clinical responses in distinct immunotherapies, tumors, and perhaps even in distinct immune-affected disorders, although we examined DC vaccine therapy for glioma exclusively.

Our findings also reveal novel IDH1 functionality. IDH1 normally plays a pivotal role in lipid metabolism and glucose-sensing within the cell, and its activity is regulated by metabolic factors, including NADP^+^, α-ketoglutarate (αKG), fatty acid biosynthesis and insulin signaling [43]. Immune-enhancing function of wild-type IDH1 has not been previously described, although our findings and others’ suggest mutant IDH1R132H inhibits T cell activity [17, 24, 25]. At least one other key sugar-metabolizing enzyme (GAPDH) is known to exhibit a non-metabolic function, but this impacts intracellular signaling rather than neighboring immune cell function [44]. Thus, our findings represent the first evidence that a classical metabolic enzyme exhibits a distinct extracellular function.

This led us to uncover a novel activity of IDH1, namely the ability to remove sialic acid from glycoproteins, including the CD8 coreceptor on T cells. Indeed, at limiting concentrations, IDH1 specifically bound to and selectively desialylated CD8 dimers. This is remarkable for multiple reasons. First, such specific immune cross-functionality is unprecedented, among both sugar-metabolizing enzymes and desialylating proteins. Second, although the sensitivity of T cell lysis to energy metabolism has long been appreciated [45, 46], our findings suggest a more direct potential link between target cell metabolism and CTL activity than previously realized. Further, our results reveal the possibility that a novel axis of T cell functional modulation exists that is relevant to cancer, but also to T cell self-reactivity in general. In any case, IDH1 released when target cells are lysed appears responsible for T cell modulation. Thus, conditions that increase target cell death, such as radiation or chemotherapy treatment of tumors, are expected to increase cell death and thereby augment IDH1-mediated T cell responses. Consistent with this, our previous studies showed that glioma vaccine therapy synergizes with chemotherapy [42]. Cell death resulting in IDH1 release could similarly impact responses to non-tumor antigens to augment or prolong normal CD8 T cell function. In this context, release of IDH1 at sites of tissue damage or by lysed targets cells could serve as a signal to maintain local T effector activity. In light of these findings, it is tempting to speculate that IDH1 represents a specific physiological regulator of T cell sialylation. CD8 sialylation on T lineage cells was previously thought to be restricted mainly to early T development, where it is regulated by the ST3Gal-I sialotransferase and other factors [12, 47]. If IDH1 is indeed able to modulate CD8 sialylation during and after T cell development, it could modulate antigen response thresholds generally, to impact selection events in the thymus, as well as tolerance and site-specific reactivity to antigens in the periphery.

In summary, our studies provide strong evidence that IDH1 expands glioma immunotherapy benefits, can enhance T cell lysis and responsiveness to multiple tumor antigens, and possesses selective binding and desialylation activity against CD8 dimers. By contrast, IDH1R132H limits immunotherapy benefits, delays T cell lysis, and both lacks direct desialylation activity and reduces that of wild-type IDH1, while retaining binding to CD8. We speculate that IDH1 may prolong anti-tumor CTL activity at sites of IDH1/NADP^+^ release by damaged tissue or killed tumor cells. Because gliomas contain substantial necrotic cell death, and also may be particularly sensitive to DC vaccine immunotherapy [48, 49], they may be especially sensitive to IDH1- or IDH1R132H-mediated modulation of local T cells in the tumor. Further study is needed to determine how to best modulate the IDH1/CD8 sialylation axis to enhance clinical success in other immunotherapies and tumors. Similarly, examining how IDH1 impacts CTL activity in non-cancerous disorders is critical to understanding its potentially broader role in modulating tissue destruction during homeostasis, aging and autoimmunity.

## Materials and methods

### Cell lines

The HTB-157.7 cell line was a generous gift from Dr. J. Schneck (Johns Hopkins Medical School, Baltimore, MD). The GL26 cell line was a gift from Dr. Henry Brem (Johns Hopkins Medical School). HTB 157.7 cells were cultured in RPMI1640/10% FBS (Invitrogen Corp.) with 1.7 mg/mL G418 (Gold Biotechnology). GL26 cells were cultured in RPMI1640/10% FBS. Authentication of the HTB157.7 was performed by verifying functional reactivity against H-2K^b^ expressing cells. Authenticity of the GL26 line was continually verified in each experiment by tumor formation upon implantation into C57BL/6 female mouse brain.

### Flow cytometry

Biotinylated PNA was obtained from Vector Laboratories. Anti-CD8-Pac Blue (53-6.7), anti-CD28 (37.51), anti-IFNγ Alexa Fluor 700 (B27), biotinylated anti-H2-K^b^ (AF6-88.5), biotinylated anti-H2-D^b^ (28–13–8), and PerCP and FITC Streptavivdin were all obtained from BD Biosciences. Cell suspensions were incubated with antibodies in PBS + 5% FBS on ice for 30 min. Cells were then washed with PBS + 5% FBS. 50,000 to 150,000 events were collected.

### Enzyme treatment of cells

10^6^ splenocytes or purified CD8^+^ cells were treated with 10 µL 1 U/mL *Vibrio cholera* neuraminidase type II (Sigma N6514), 0.05 mg IDH1 or IDH1R132H with or without 100 µM NADP^+^ for desiaylation experiments. 5.5 μL of 500 mM CMP-sialic acid was added to every 7.5 µL ST3Gal-II per 10^6^ cells to sialylate. Cells were incubated in 1 mL RPMI/10% FBS for 30 min at 37 °C, after which they were rinsed and stained.

### Multimer stimulation

The following MHC dextramers were purchased from Immudex: H-2D^b^/ATFKNWPFL (Survivin), H-2K^b^/SVYDFFVWL (Trp-2), H-2K^b^/TVSEFLKL (Survivin), H-2K^b^/SDYYFSWL (muFAPα), H-2K^b^/HILIYSDV (MYBPC-2), H-2K^d^/TYLPTNASL (HER2), and H-2K^d^/QYIHSANVL (ERK1). HLA-A*02:01/KVLEYVIKV (MAGE-A1) was purchased from Beckman Coulter. Additional MHC tetramers (Beckman Coulter) were used for in vitro stimulation: H-2D^b^/KVNPRNQDWL (Hugp100); H-2K^b^/WVYDFFVWL (Trp-2); H-2D^b^/KAVYNFAT (LCMV); H-2K^b^/SIINFEKL (Ova); HLA-A2/empty. Splenocytes or CD8 T cells were stimulated for 2, 6, or 10 h with multimer and anti-CD28; golgi plug (BD Biosciences) was subsequently added. Cells were then permeabilized and stained.

### FITC conjugation

IDH1 was conjugated to FITC by adding 5 μL of 1 mg/mL FITC per 1 mL of 2 mg/mL protein solution. After protein-FITC conjugation, unconjugated FITC was removed by dialyzing against PBS using Snake Skin Dialysis Membrane.

### Sialidase activity of IDH1

Sialidase activity assays were performed using the Amplex Red Neuraminidase Assay Kit (Life Technologies). 4 µg (high concentration) or 1μg (low concentration) purified IDH1 and IDH1R132H were incubated with and without 2 µg CD8α, CD8β, CD8αβ, or fetuin, and 100 µM NADP^+^ for one hour at 37 °C in reaction buffer (0.05 M Tris-HCl, pH 7.2, 1 mM CaCl_2_). N-20 (anti-IDH1; Santa Cruz Biotech), or isotype-matched goat IgG, was used for antibody adsorption of lysates as described. Plates were read on a spectrophotometer at 560 nm every 5 min. Percent of neuraminidase activity was calculated as [(enzyme with substrate − no substrate control)/VCN positive control] × 100.

### Coimmunoprecipitation and western blots

Splenocyte cell surfaces were biotinylated using NHS biotin (Thermo Scientific). Samples were then lysed with cell lysis buffer (Cell Signaling Technologies) containing a complete protease inhibitor (Roche). Lysed samples were precleared with goat or mouse IgG 3 times, and then probed with IDH1 or IDH1R132H for 30 min. IDH1 and IDH1R132H were removed by incubating with their respective antibodies and then pulled down with protein G agarose beads. Bead pellets were washed 3 times and then boiled. Western blots were performed as described previously [10], using anti-CD8α (2.43; ATCC) or isotype-matched control rat IgG2b at 1:200 as primary, goat-anti-rat-HRP at 1:500 as secondary, and developed via chemoluminescence.

### LDH release

LDH release assays were performed using a Cytotoxicity Detection Kit (Roche Applied Science) according to manufacturer’s instructions. 25,000 target cells were incubated with 250,000 effector cells for either 4, 7, or 14 hours at 37 °C. For imidazole treatments, cells were treated with 100 mM or 200 mM imidazole and then incubated for 4 hours at 37 °C. Cytotoxicity was calculated as [(Target cell − negative control)/(positive control − negative control)] × 100. Vehicle control or 5 mM oxalomalic acid (OMA) was added to triplicate wells for inhibition studies. For antibody blocking, 1 μg of N-20 (anti-IDH1; Santa Cruz Biotech), or isotype-matched goat IgG, was added to triplicate wells. Cytotoxicity was calculated as [(effector, target cell mix − effector control) − negative control/(positive control − negative control)] × 100.

### Animal studies

Female C57BL/6 mice were purchased from Jackson Labs, and female B6-Foxn1 mice were purchased from Harlan Inc. Mice were housed in a pathogen–free vivarium. Tumor implantation and DC vaccine preparation were done as described previously [19]. Mice were vaccinated subcutaneously 3, 7, and 10 days post tumor implantation with 50,000 (normal dose) or 2 × 10^7^ (high dose) GL26 lysate-pulsed, irradiated DC 2.4 cells. Mice were euthanized upon acquisition of terminal symptoms.

### TCGA data analysis

Gene expression data from TCGA for low grade glioma and glioblastoma was downloaded through cBioPortal (www.cbioportal.org) [50, 51]. Gene expression data was stratified by IDH1 WT or IDH1R132H status. All other IDH1 and IDH2 mutations were excluded from the analysis.

### Patients & treatment

#### Patient data analysis

All patient data came from an ongoing Phase I clinical trial (NCT01792505). In the analysis of the patient data, a single IDH1WT patient was excluded due to their unique non-mutated p53 status.

#### DC vaccination

DCs were prepared according to our established protocol [1]. DCs were washed three times in PBS, resuspended at 10^6^ cells/ml in complete media, and then coincubated with autologous tumor lysate (100 μg/ml) for 16–20 h.

Imiquimod cream (5%, 250 mg) was self-applied topically by patients to a 4 × 5-cm outlined area of healthy skin in the axillary region overnight on days 1–5 of each cycle. Dendritic cells were injected intradermally into the Imiquimod-treated site on day 3. Cycles were repeated every 2 weeks for a total of three injections.

#### ELISPOT

ELISPOTS were performed using BD ELISPOT plates and sent out to a third party facility for automatic quantification on an ELISPOT plate reader.

### Docking studies

The template crystal structures for IDH1 and IDH1R132H used for docking studies were taken from 1T09 [52]. The template for sialic acid was taken from the crystal structure of viral neuraminidase 4GZQ [53]. Solvents, isocitrate and Ca^2+^ were removed from the IDH1 crystal structure The UCSF Chimera program was used to prepare the enzyme for docking studies. Docking studies for isocitrate and sialic acid into IDH1, as well as Ki and Gibbs free energy calculations were performed using Autodock. Images were created using Pymol software.

### Statistics

The Student’s *T* test was used to measure statistical difference between experimental groups. The Log-Rank test was used for survival studies. All error bars represent ±SEM. *P* < 0.05 was considered significant.

### Study approval

All animal procedures were approved by the Cedars-Sinai Medical Center Institutional Animal Care and Use Committee. All patients involved in the clinical trial NCT01792505 gave their written informed consent. Inclusion criteria, exclusion criteria, and study design of the clinical trial were approved by the Cedars-Sinai Medical Center Institutional Review Board.

## Supplementary information

Supporting information

## Data Availability

Results and raw data will be made available upon request. Model Organisms and/or the means to generate them will be made generally available for research (non-commercial) use.
