# Supplementary material for: Glioma immunotherapy enhancement and CD8-specific sialic acid cleavage by isocitrate dehydrogenase (IDH)-1
Source: Oncogene. 2023 May 9;42(25):2088–98. doi: 10.1038/s41388-023-02713-7 (PMC10275753; doi:10.1038/s41388-023-02713-7)
Supplement: Supplementary file 1 — Supporting information [file 41388_2023_2713_MOESM1_ESM.pdf]

Supporting Information for

Glioma immunotherapy enhancement and CD8-specific sialic acid cleavage by Isocitrate Dehyrogenase (IDH)-1

Authors:

Ryan Cordner, Michelle Jhun, Akanksha Panwar, HongQiang Wang, Nicole Yeager, Ramachandran Murali, Joseph H. McAbee, Armen Mardiros, Akane Takei, Mia W. Mazer, Xuemo Fan, Emmanuel Jouanneau, John S. Yu, Keith L. Black,

and Christopher J. Wheeler

correspondence to: chris.wheeler@brainmappingfoundation.org; chris@tneuropharma.com

This file includes:

Figs. S1 to S11

Supplementary Figures

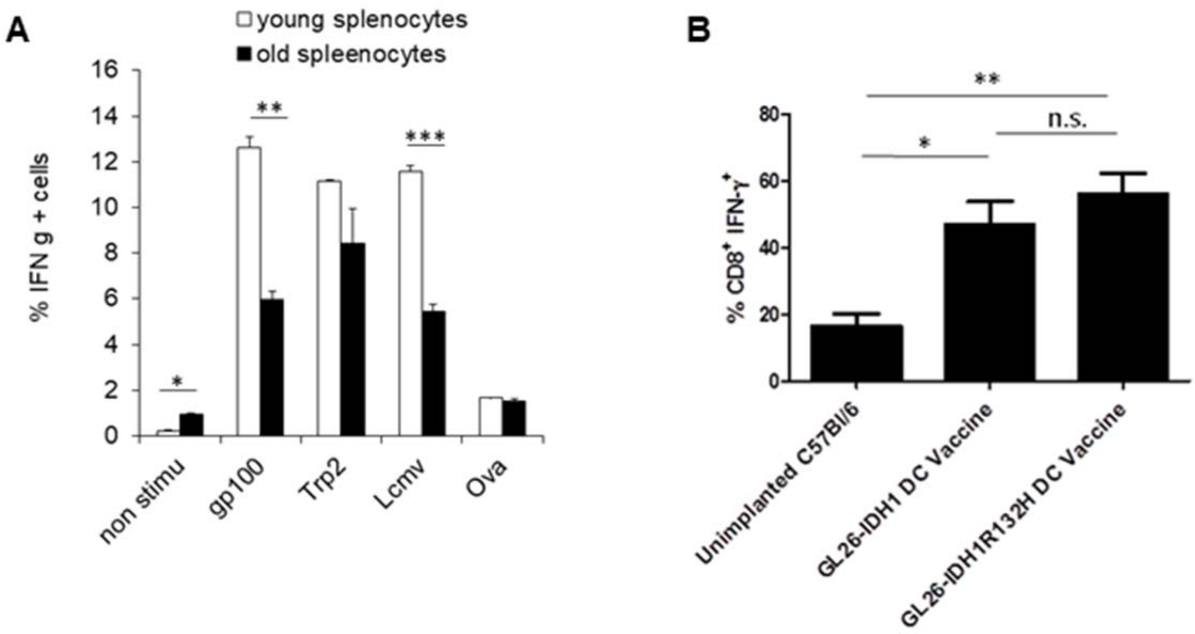

Fig. S1 (A) Spleen cells from young (1-2 week old pups) and old (12 months) B6 mice were stimulated with the indicated pMHC-I multimers + anti-CD28 mAb as previously described (Jouanneau et al., Cancer Immunol Immunother 63:911-924), and percent IFNγ^{+} CD8 T cells quantified by flow cytometry. (B) Peripheral immune response in GL26 implanted mice. CD8 T cells from the spleens of tumor implanted or unimplanted B6 mice were stimulated with Trp-2 MHC tetramers. The percentage of IFN-γ producing $CD8^{+}$ cells was quantified using flow cytometry. *p<0.05; **p<0.01; ***p<0.005.

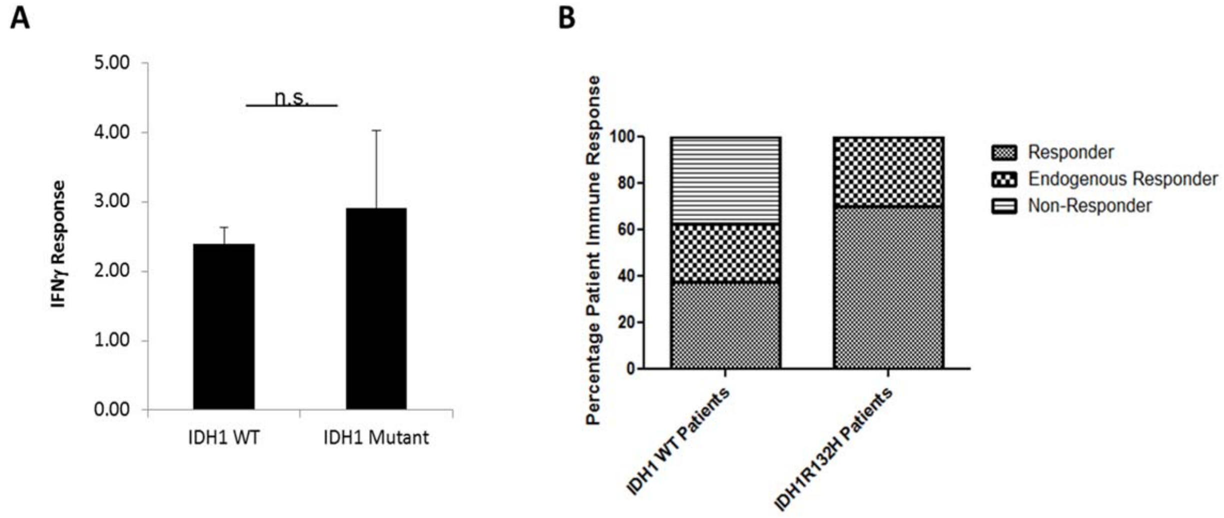

Fig. S2 Peripheral immune response in IDH1-mutated GBM patients. (A) IDH1 WT and IDH1 Mutant patients showed similar peripheral IFN-γ responses after DC vaccination. IFN-γ responses were assessed by flow cytometry as described in methods. (B) Patient peripheral blood mononuclear cells were stimulated with autologous tumor lysate. Patients producing ≥50% more IFN-γ after vaccination were considered to be immunological responders. Endogenous responders were those patients that already exhibited an IFN-γ response prior to vaccination, and this response was not increased by vaccination. Patients that did not produce IFN-γ in response to vaccination were considered to be immunological non-responders.

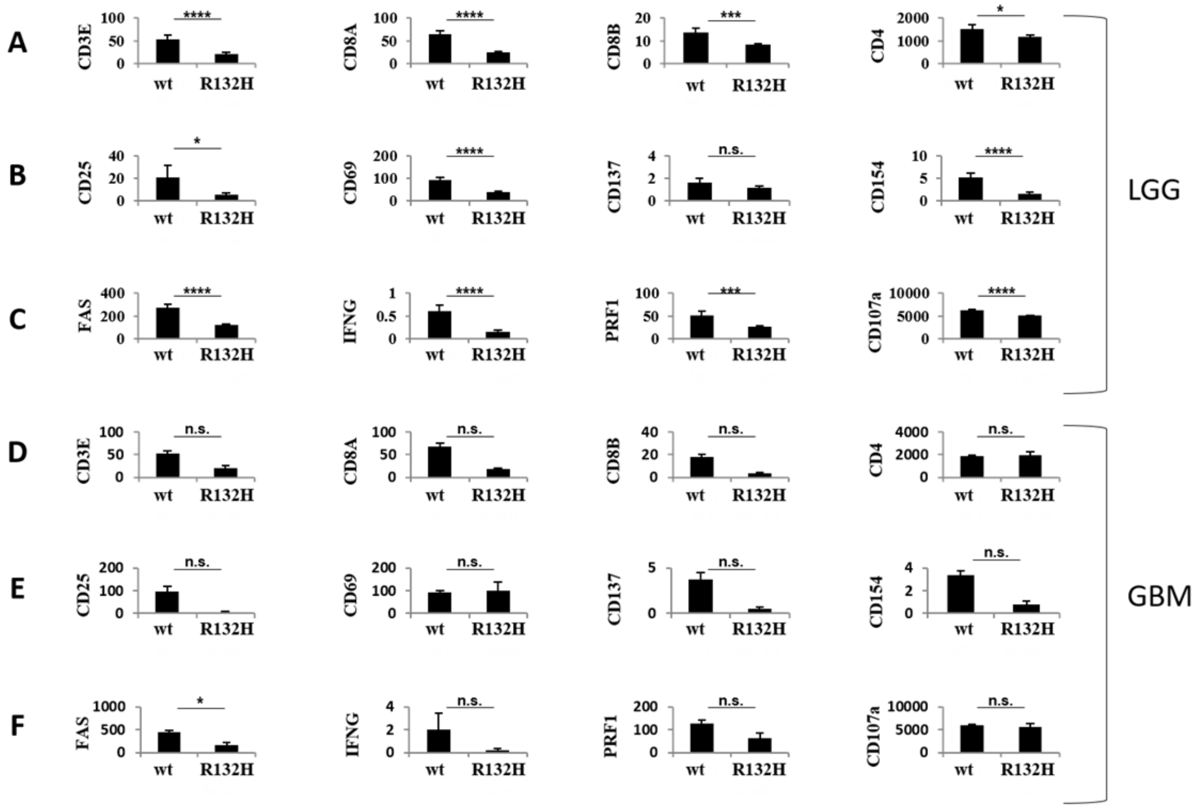

Fig. S3 CD8 T cell gene expression in IDH1 mutated low grade glioma and glioblastoma. (A) Gene expression data from The Cancer Genome Atlas (TCGA) on low grade gliomas shows markedly decreased expression of CD8 T cell specific genes in tumors with IDH1 mutations. (B) Gene expression of three out of four T cell activation markers, and all T cell lytic genes interrogated (C) was significantly decreased in IDH1-mutated tumors. (D) Similar decreases were observed in CD8-specific T cell genes, and in CD8 T cell activation markers (E), in IDH1-mutated GBM tumors, but failed to reach statistical significance, likely due to the small number of IDH1 mutated tumors in TCGA. (F) A significant decrease in Fas expression was observed in IDH1-mutated GBM, but was not evident for other lytic effector genes. For low grade glioma, IDH1 WT n=51, IDH1 Mutant n=218. For GBM, IDH1 WT n=129, IDH1 Mutant n=7. *$p<0.05$ **$p<0.01$ ***$p<0.005$ ****$p<0.001$.

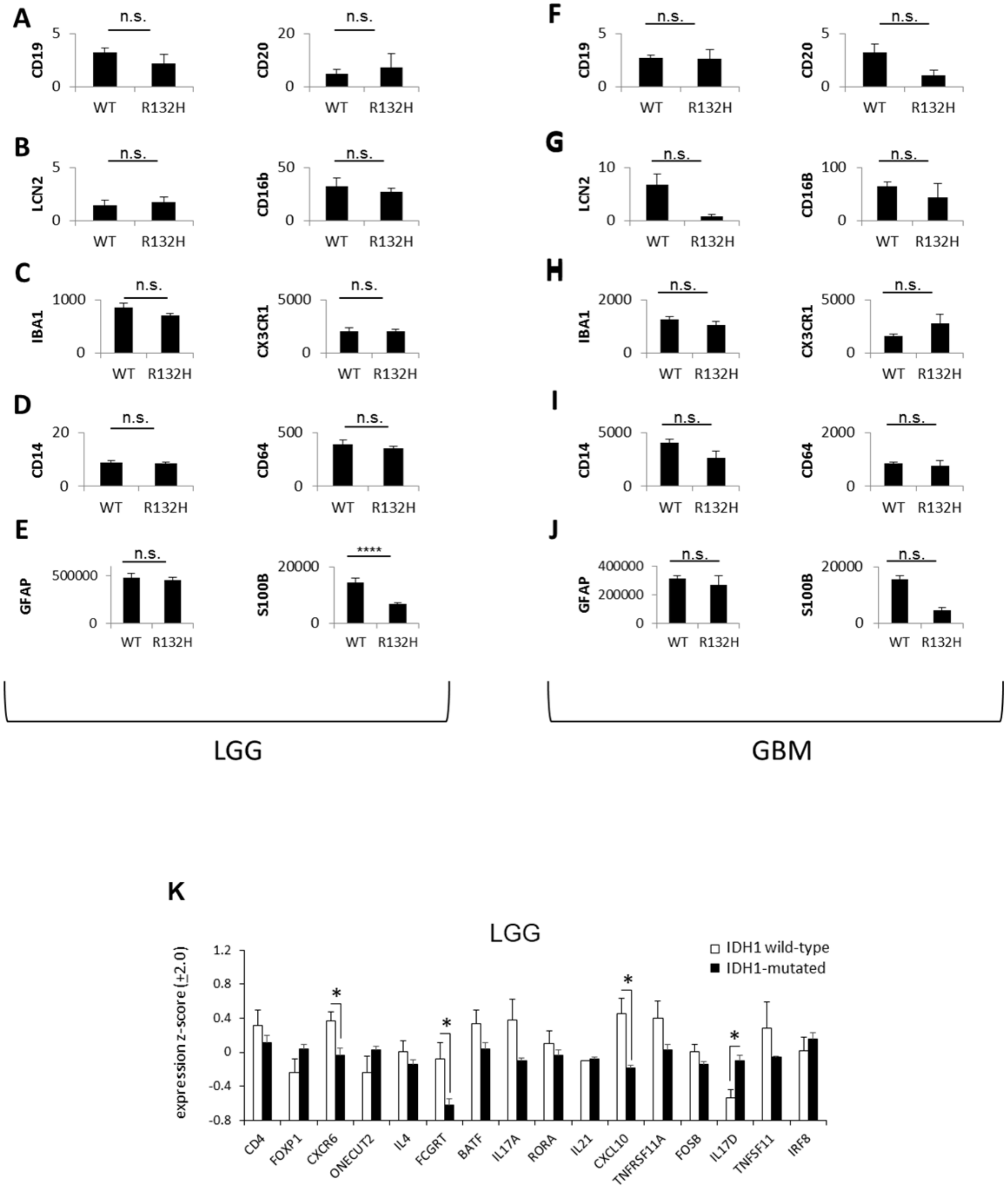

Fig. S4 Similar Innate and adaptive immunity gene expression from TCGA tumors. (A-E) Low grade gliomas show similar expression of innate and adaptive immunity genes. (F-J) Glioblastoma tumors show similar non-significant trends as the low grade glioma data. (K) Low grade gliomas show minimal

alterations in CD4 and T-helper subset-defining genes with IDH1 mutation. IDH1 WT n=51, IDH1 Mutant n=218; *p<0.05.

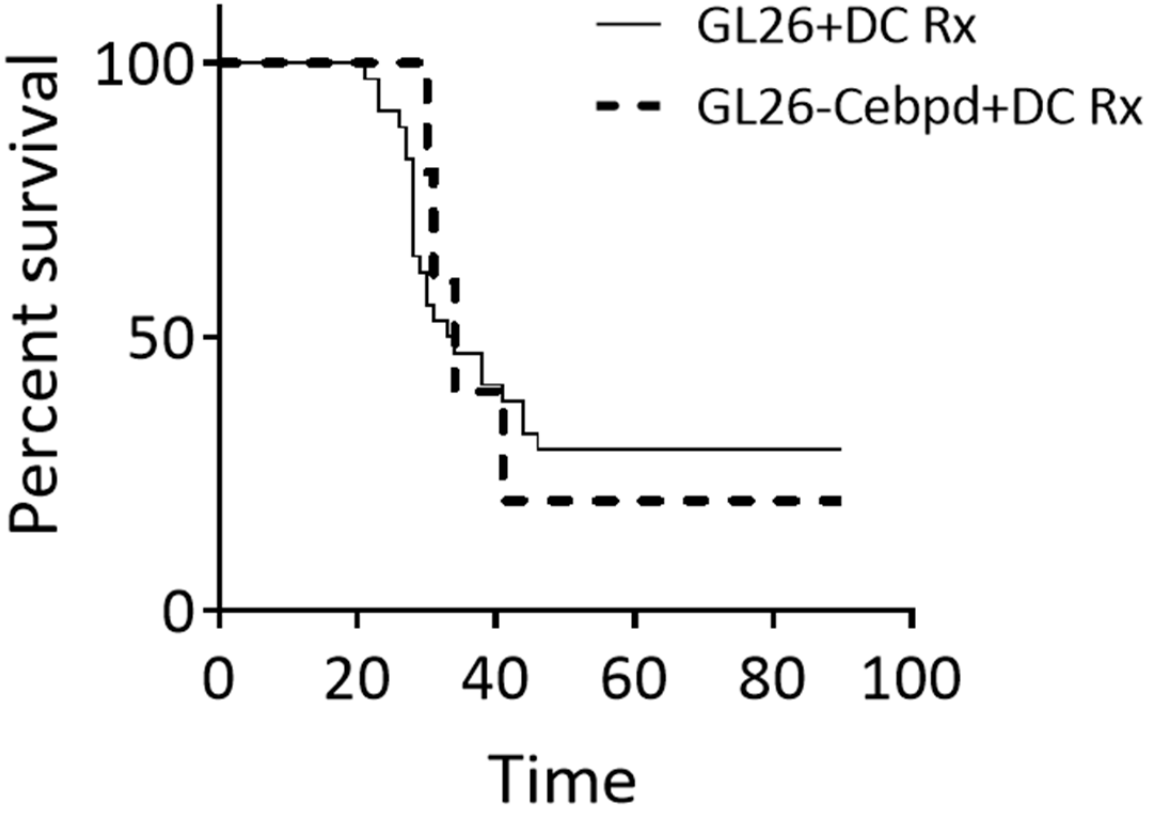

Fig. S5 Kaplan-Meier plot comparing survival of untransfected GL26 (also in Fig. 2B) to control (Cebpδ)-transfected G:26 after therapeutic DC vaccination (n = 5; median 33.5 vs. 34 days; *P* = 0.989, Log-rank). There was also a trend toward distinct survival times in control-transfected vs. IDH1-transfected GL26 after DC vaccination (*P* = 0.09, Log-rank).

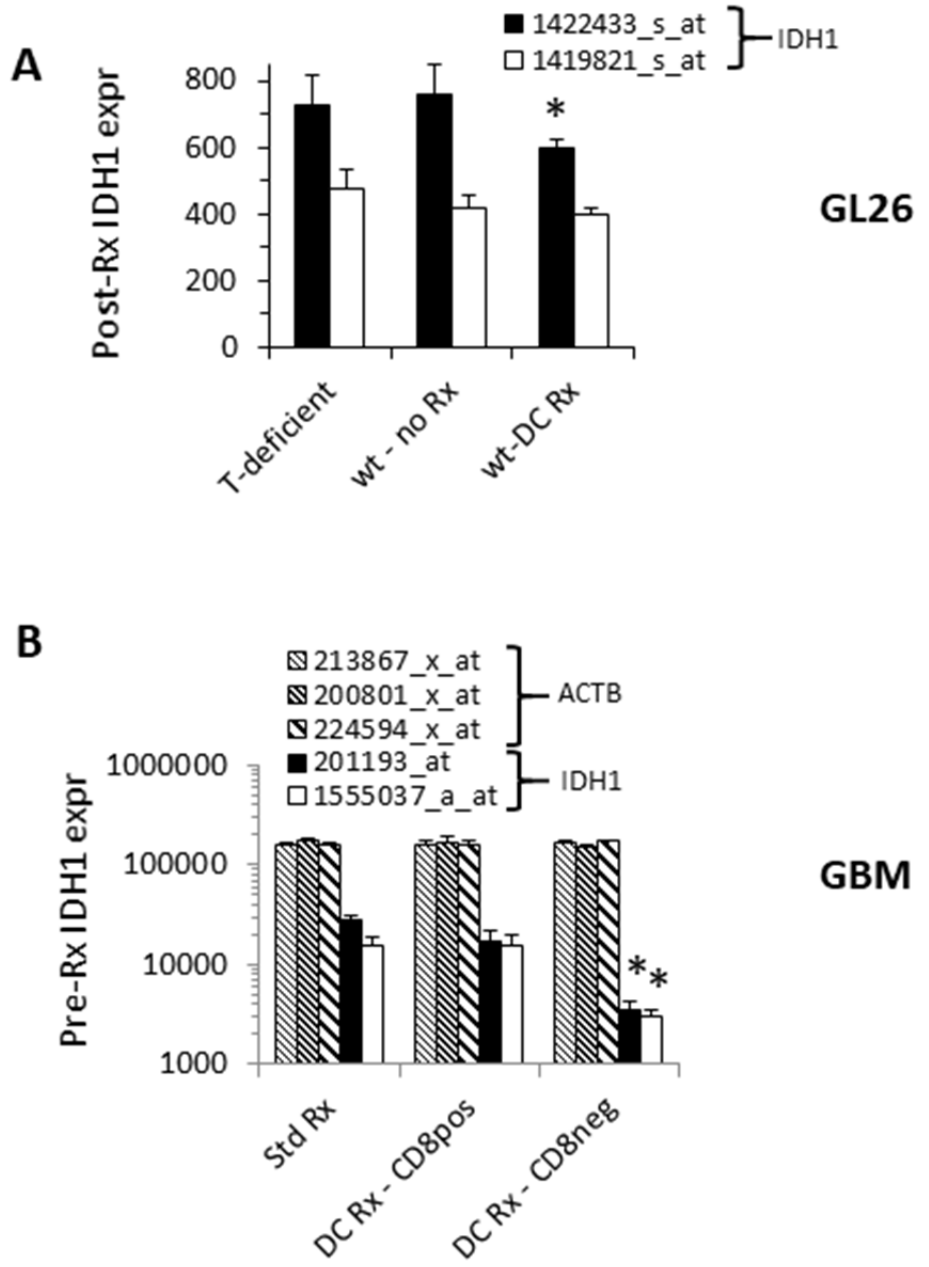

Fig. S6 (A) RNA from GL26 tumors (n $\geq$ 4) growing in brains of terminally symptomatic C57BL/6 (B6) mice with and without DC vaccine treatment, or in those of untreated T cell-deficient mice (B6.Foxn1), were subjected to expression analysis using MG-430 2.0 microarray chips. Normalized IDH1 expression was significantly lower in GL26 from DC vaccinated wild-type mice than in either T cell-deficient or untreated B6 hosts. (B) Pre- and post-therapy RNA from conventionally-treated GBM, and from DC-vaccinated GBM with and without evidence of intratumoral T cell response (significant vs. no increase in CD8 signal), was similarly analyzed using HG-U133 plus 2 microarray chips. IDH1 probesets with relative signal >10000 are shown, along with normalized controls (ACTB). IDH1 was significantly lower in CD8-negative vaccine cohort (n = 3 vs. n = 7; *P < 0.03, 1-sided T-test).

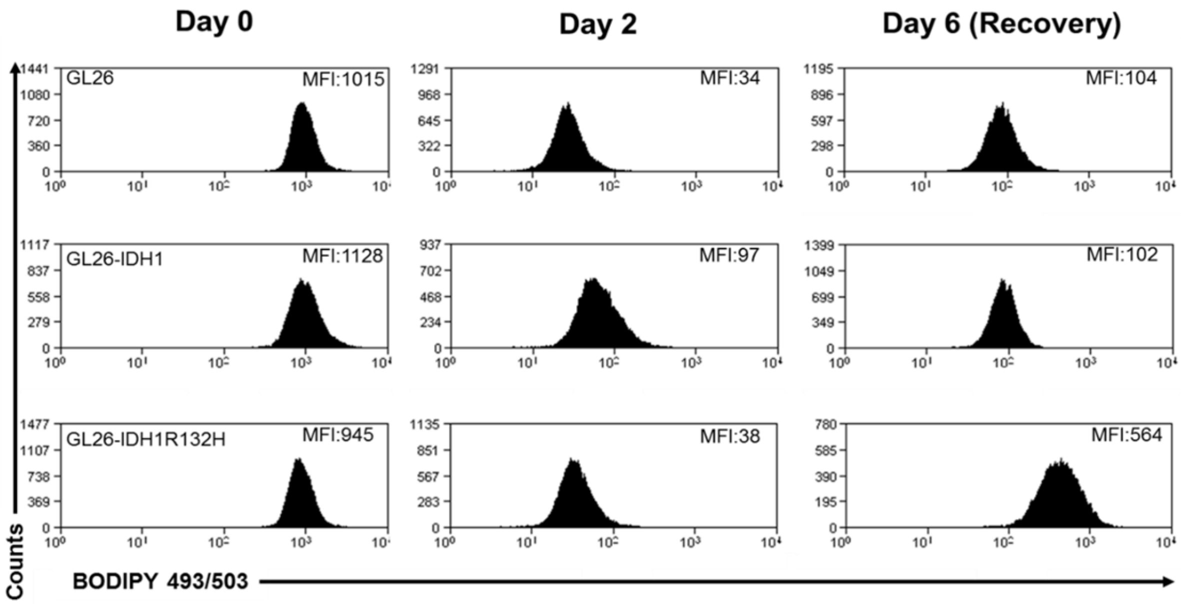

Fig. S7 IDH1 and IDH1R132H transfection alters lipogenesis. 250,000 GL26, GL26-IDH1, and GL26-IDH1R132H cells were cultured in glucose free, glutamine rich (4mM) RPMI 1640 for four days. Glucose rich (11mM) RPMI 1640 was restored on day 4 and cells were allowed to recover for two days. Cellular lipids were assessed with the neutral lipid stain BODIPY 493/503 on days 0, 2, and 6. GL26-IDH1R132H showed altered lipogenesis with an enhanced recovery of cellular lipids after glucose restoration.

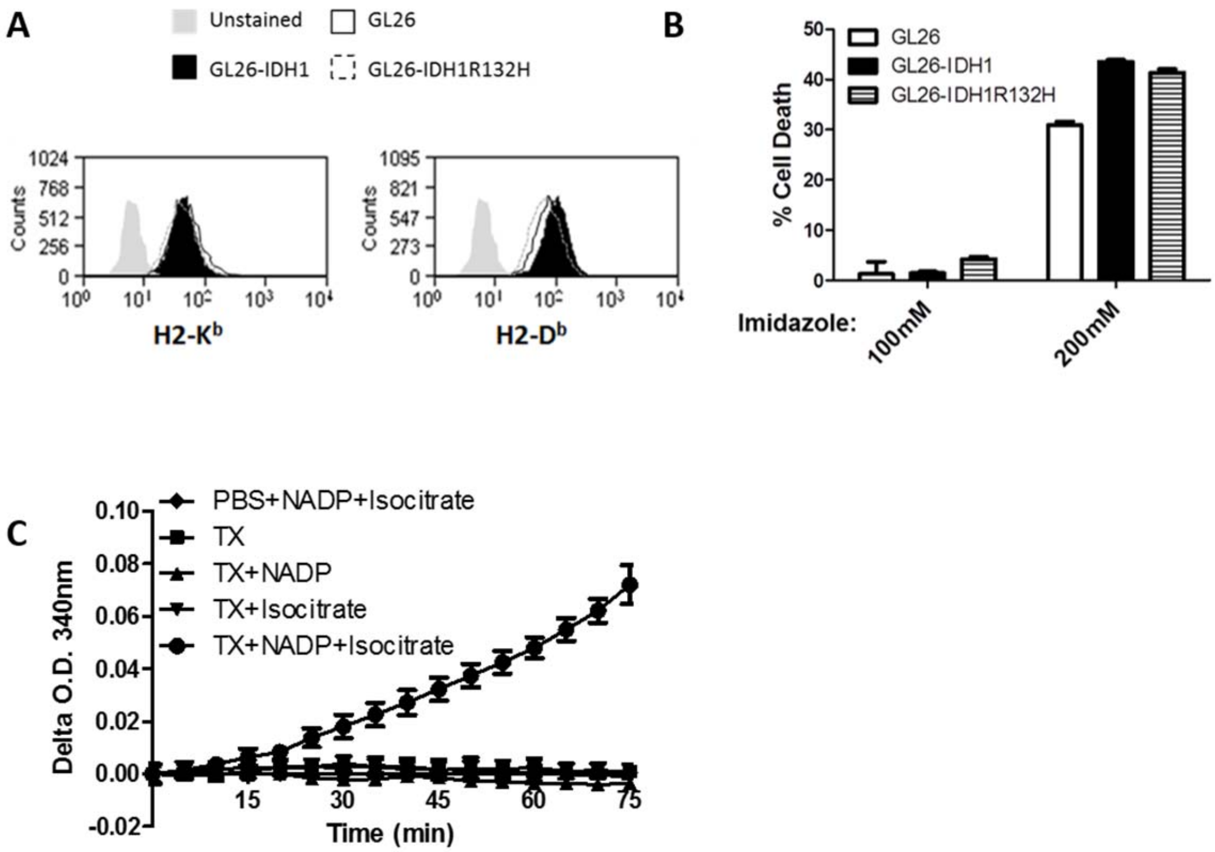

Fig. S8 Neither IDH1 overexpression nor IDH1R132H alters target antigen or death sensitivity, and enzymatically active IDH1 is released from lysed tumor cells. (A) Flow cytometry verified identical MHC I expression in each of the GL26 sub-line used for cytotoxicity assays. Representative histograms are shown. (B) GL26 glioma cell lines used for cytotoxicity assays did not exhibit an intrinsic resistance to apoptosis when treated with 100 or 200mM imidazole. (C) HT22 neuronal tumor cells were incubated for the indicated times with 0.1% Triton X-100 (TX) or PBS with and without $NADP^+$ and/or isocitrate, monitoring NAPDH production by spectrophotomety.

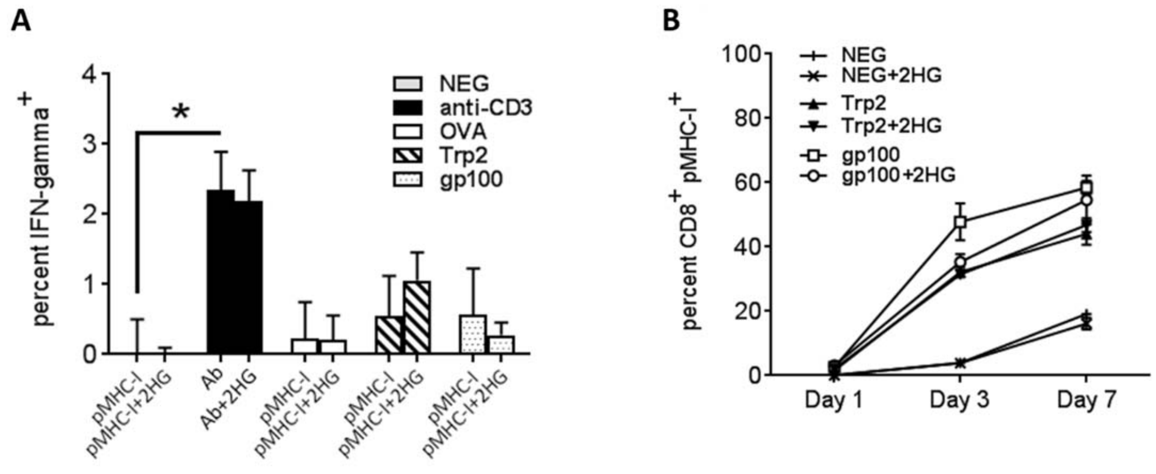

Fig. S9 (A) Extracellular 2-hydroxyglutarate (2HG) does not impair CD8 T cell function. T cells were purified from naïve C57BL/6 donors by MACS affinity column, and stimulated with either indicated pMHC-I tetramers (Beckman Coulter) or anti-CD3ε, plus anti-CD28 antibody (BD Biosciences). Proportions of viable $CD8^+$ cells co-expressing IFNγ were quantified after 10 hours stimulation in 3 independent replicates. (B) Extracellular 2HG had no significant impact on antigen- or anti-CD3ε-mediated CD8 T cell proliferation up to 7 days after stimulation. 2HG was added at 10mM; tetramers and antibodies for stimulation were added at 1μl and 1 μg/50,000 viable cells in 150 μl volume RPMI-1640/10, respectively. Percentage IFN-γ in unstimulated control CD8 T cells was subtracted from all values prior to plotting.

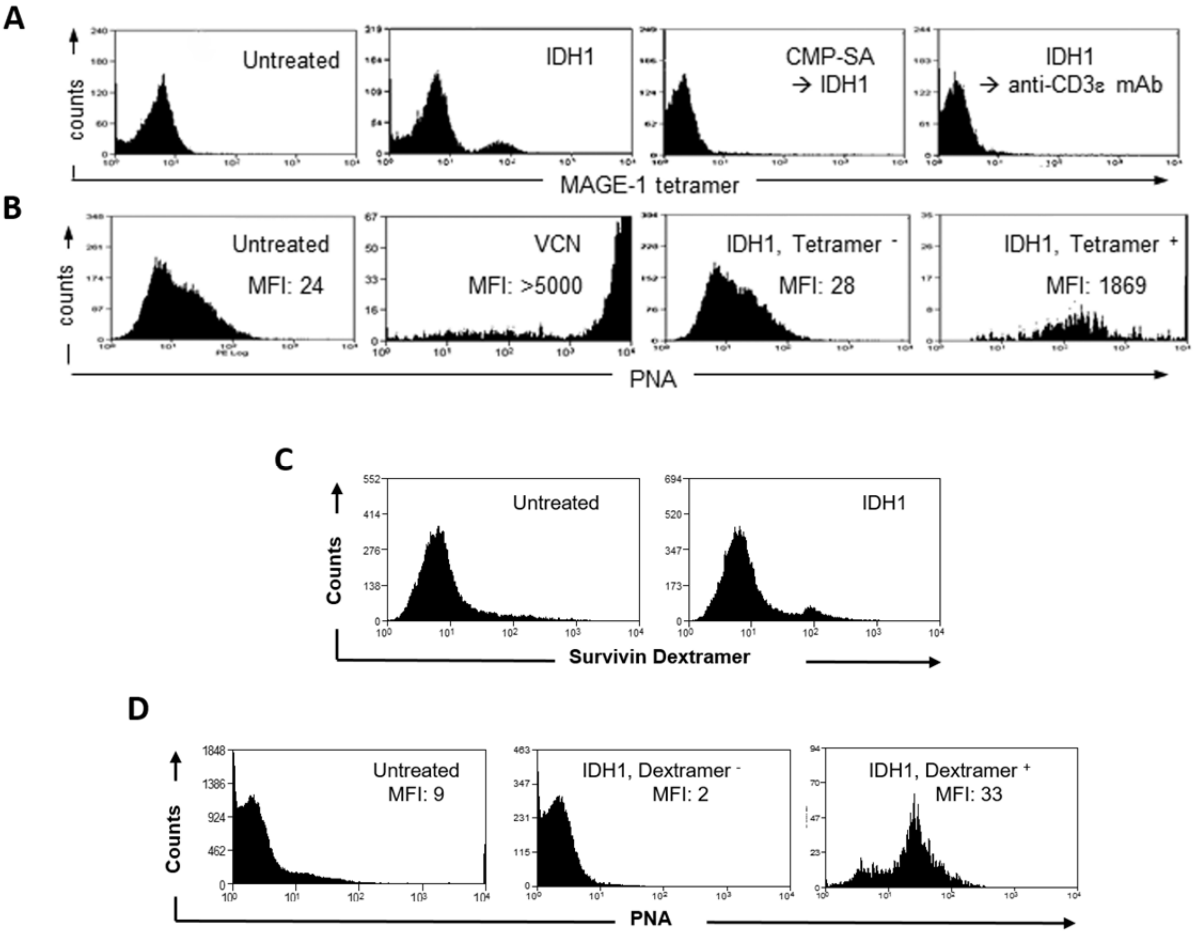

Fig. S10 (A) IDH1 treatment increases binding of human CD8 T cells to HLA/MAGE-1 tetramers, and is blocked by addition of free CMP-sialic acid prior to, or anti-CD3ε antibody following, IDH1 treatment. (B) Human CD8 T cells treated with *V. cholera* neuraminidase (VCN) displayed increased PNA binding on all cells, whereas treatment with IDH1 increased PNA binding only on MAGE-1 tetramer^{+} cells. IDH1 treated mouse CD8^{+} cells display higher dextramer binding. (C) IDH1 treatment increases mouse CD8+ cell binding to H-2K^{b}/TVSEFLKL Survivin dextramer. (D) Survivin dextramer positive cells display selective desialylation by PNA binding.

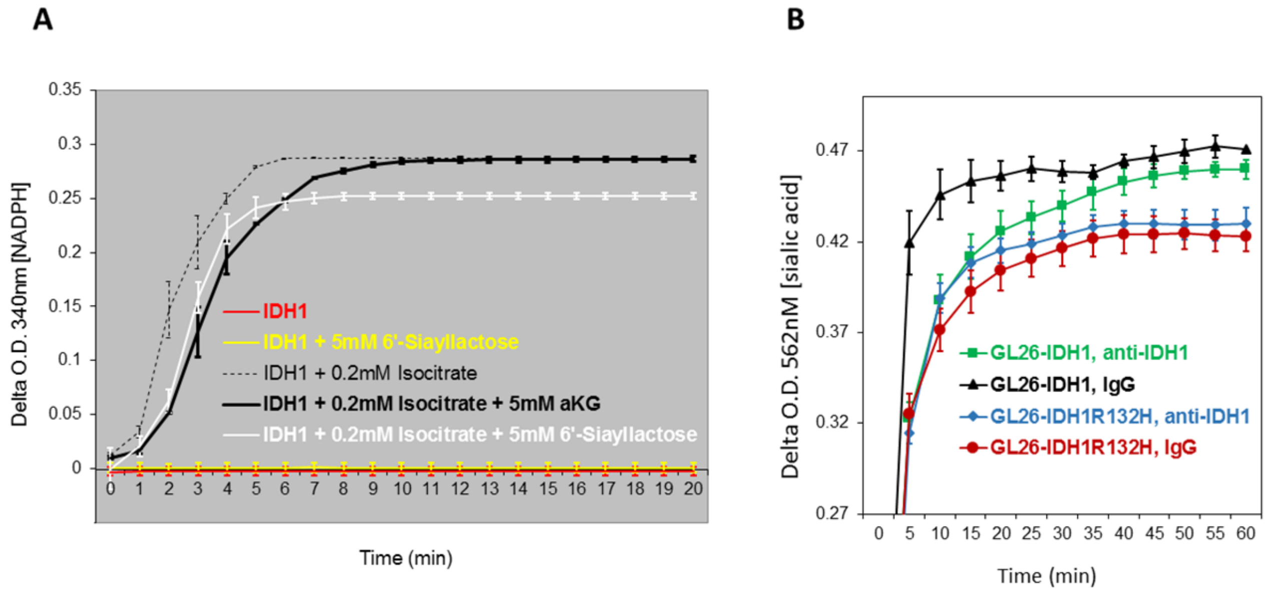

Fig. S11 (A) Inhibition of established IDH1 enzymatic activity by a sialylated glycan: 11 mg/ml IDH1 was incubated with 1.92mM $NADP^+$, 100mM $CaCl_2$, and 0.2mM isocitrate with and without the addition of 5mM α-ketoglutarate (aKG; a known inhibitor of IDH classical enzymatic activity) or 6'-sialyllactose. Reactions were incubated at 18°C, and NADPH production monitored each minute by the increase in OD at 340nm. Reactions with aKG or 6'-sialyllactose were significantly different than the control IDH1 + isocitrate reaction (both $P < 0.0001$ by Two-way ANOVA). (B) Reduction of tumor lysate-derived sialidase activity by anti-IDH1 antibody and IDH1R132H: 500 μl each of GL26-IDH1 and GL16-IDH1R132H lysates (5x10^7 cell equivalents @ 0.57 μg/ml) was subjected to two rounds of incubation with 1:50 anti-IDH1 or control antibody (rabbit anti-goat IgG; both 0.5 mg/ml) 1 hr at 4°C, followed by Protein G-agarose 30 min at 4°C, centrifugation, and retention of absorbed supernatants. Sialic acid (SA) release from fetuin substrate was monitored every 5 minutes by the increase in OD at 562nm. SA production with anti-IDH1 was significantly different than with IgG in GL26-IDH1 lysate only, and between IgG-absorbed GL26-IDH1 and GL26-IDH1R132H lysates ($P < 0.005$ and < 0.0001 by Two-way ANOVA, respectively).
